# The relationships of job and family demands and job and family resources with family caregivers’ strain

**DOI:** 10.1111/scs.12873

**Published:** 2020-05-12

**Authors:** Nicolle P. G. Boumans, Elisabeth Dorant

**Affiliations:** ^1^ Department of Social Medicine Maastricht University Maastricht the Netherlands

**Keywords:** family caregiving, paid employment, job demands, family demands, job resources, family resources, work–family and family–work conflict, work–family and family–work enrichment

## Abstract

**Background:**

Because of an expected increase in the number of family caregivers, there is a growing public and scientific interest in family caregiving and more specifically in the combination of family care with paid employment. It is important to gain insight in the family caregivers’ strain and determining factors in the job and family domain.

**Aim:**

The aim of the study was to examine the associations of job and family demands and job and family resources with indicators of caregivers’ psychological strain, that is caregiver burden, work‐related emotional exhaustion and general ill mental health. In our research, we focused on individuals who combine paid employment with family caregiving.

**Methods:**

A cross‐sectional design was used. The study sample was derived in 2011 from a Dutch financial organisation and a healthcare organisation. A digital fully structured questionnaire was used. The sample consisted of 187 employees who identified themselves as family caregivers. Descriptive statistics and hierarchical linear regression analysis were performed.

**Results:**

Job demands (i.e. workload, work–family conflict) and family demands (i.e. family care hours and family–work conflict) were significantly positively associated with all three domain‐specific indicators of strain. The resources of work–family and family–work enrichment and autonomy did not contribute to less experienced strain. More supervisor and colleague support was associated with lower ill mental health.

**Conclusion:**

Our study showed that job demands (workload, work–family conflict) and family demands (family care hours, family–work conflict) were clearly associated with caregiver strain, while associations for job and family resources were not evident. It remains necessary to pay attention to the demanding aspects of dual roles of family caregivers but also to investigate the resources they have available at work as well as in their home situation and explore their potential reducing effect on family caregivers’ strain.

## Introduction

Family caregiving involves caring for a chronically ill or handicapped partner, parent, child or other family member or friend in need, without any financial compensation (1, 2), and is a level of care that exceeds the usual day‐to‐day care for that person (3).

Family caregivers are responsible for a substantial part of the care for relatives and friends in need of care at home (4). Actually, family and friends provide more care than professional health carers, but, surprisingly, its worth is often overlooked (5). In the Netherlands (2007), the monetary value of family care of adults who provide help was estimated at € 6.6 billion (6).

In European countries, the number of family carers is increasing because of multiple societal changes, healthcare advances and changes in long‐term care policies (7). For the Netherlands, predictions are that the reform of long‐term care in 2015, which entailed cutbacks in professional home and residential care and a reduction in the accessibility of professional long‐term care, will lead to more persons providing family care because of a rise in people in need of care at home (8, 9).

Although some studies reported positive consequences of family caregiving (e.g. satisfaction, emotional rewards, personal growth, competence, mastery and increased faith and spiritual growth) or of the dynamics between the caregiver and care receiver (relationship gains, satisfaction in reciprocity and fulfilling a sense of duty) (10, 11, 12), a myriad of studies have been published on adverse outcomes, such as poor mental health (e.g. 13, 14, 15), lower rates of well‐being (e.g. 16), caregiver burden (e.g. 17) or poor physical health (e.g. 18). According to figures from Statistics Netherlands (CBS Statline 2016), one in seven family caregivers in the Netherlands feels overloaded (19).

Ageing of the population contributes to increased need for more high‐intensity support, defined as care for at least eight hours per week and for at least three months (3), especially among those elderly who suffer from long‐term and complex health problems (7). Data from the European Social Survey (ESS), collected in 2014 in 20 European countries, showed that on average 34.3% of the total population were family caregivers and 7.6% were high‐intensity caregivers. For the Netherlands, these rates were, respectively, 37.3% and 6.11% (20). Other figures, from 2016, showed that about 32% of the Dutch population over 16 years (4 million inhabitants) provided family care, with about 17% caring for a family member or friend for longer than three months and for more than eight hours per week (3).

To keep up with increasing care demands, a significant number of family members are obliged to combine paid employment with care for relatives. The ESS reported that 50.9% of the carers was combining paid employment with family caregiving (20). In the Netherlands, in 2016, about 68% of the citizens of 16 and older who provided care held a paid job (3).

Employees with family caregiving responsibilities are susceptible to adverse consequences, especially when they have to juggle with demands from both the work environment and the home environment. In a Dutch study, 19% of the employed family caregivers felt overburdened by their caregiving responsibilities (21). Figures from the 2013 US National Health and Wellness Survey demonstrated lower levels of mental and physical health, and higher levels of depression and anxiety among employed family caregivers compared to employed noncaregivers (22). In addition, higher levels of caregiver burden (23), emotional strain (24) and time pressures in the work environment (25) have been reported. So‐called work–family conflict, or vice versa family–work conflict, arises when the role demands stemming from one domain (work or family) are incompatible with role demands stemming from the other domain (family or work) (26). The role stress theory, which theorises how managing multiple roles creates strain through depletion of resources (26, 27) provides an explanation for these adverse health effects. In 1992, Frone et al. were the first to demonstrate the two directions in the inter‐role conflict: work to family and family to work. In his ‘cross‐domain’ hypothesis, different stressors and consequences are of interest, and problems in the one domain, work or family, cross over to the other domain and negatively influence the performance in that domain (28). On the other hand, a ‘matching‐hypothesis’, which assumes that the primary effect of work–family conflict lies in the domain where the conflict originates and does not cross over, has also been proposed (29, 30).

Combining multiple roles and demands from both domains not automatically results in feelings of strain, especially not when caregivers can fall back on sufficient resources (31). The work–family enrichment theory postulates that resources generated in one domain or role promote higher performance and positive affect in the other domain or role (32). Work and family resources, such as social support, can enable employees to blend family caregiving tasks with formal employment successfully (33, 34). The job demands–resources (JDR) model, a prominent theory in the occupational health psychology, underscores the interplay between demands and resources in determining strain outcomes (35).

When studying employed family caregivers’ outcomes associated with difficulties in combining work with care, it makes sense to consider both demands as well as resources in both work and home situations as important determinants of strain (30). However, surprisingly little is known about domain‐specific demands and resources for this vulnerable group. Some studies exclusively concentrated on the demanding roles in both work and family in relation to caregiver strain, for example by focussing on the relationships between caregiving demands, job demands and caregiver health outcomes (36), while others only investigated various types of resources without taking the demands into account (e.g. 37). The few publications we found that made the distinction between work and family caregiving demands and resources reported an increase in caregiver strain with demanding aspects of caregiving and work responsibilities, while the use of resources in both domains helped to reduce negative experiences of stress (34, 38).

**Figure 1:**
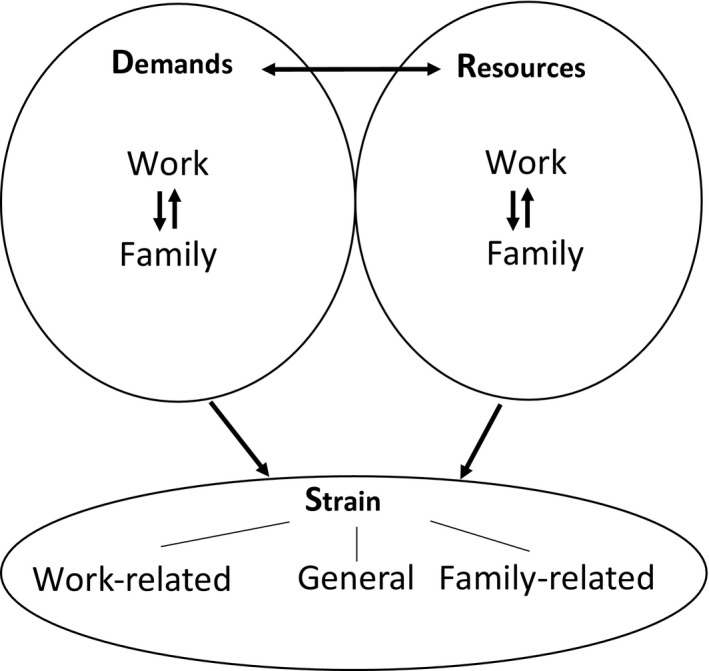
Conceptual model to study associations between demands and resources, and bidirectional conflict and enrichment in the work and family domain, with three indicators of strain in employed family caregivers.

The goal of this study is to examine the associations between demands and resources in two domains, work and family, with indicators of strain, for family caregivers with a paid job. The conceptual framework (Fig. 1) incorporates both work and family demands and resources, bidirectional conflict and enrichment variables, and their relationships with three domain‐specific indicators of strain. By gaining more insight into these associations, we hope to contribute to prevention of caregivers’ strain often linked with difficulties in combining work with care.

## Methods

### Design

In this cross‐sectional study, data were collected by a self‐administered digital questionnaire.

### Participants

Participants were recruited from a Dutch financial organisation in the private sector, delivering financial services in the field of insurances and pensions, and from a healthcare organisation, providing intra‐ and extramural healthcare services for people of all ages who require care.

Data on work‐related and family care‐related issues were collected by a self‐administered digital questionnaire in 2011. All employees of both companies were invited to participate via email. After informed consent, participants received the questionnaire and were asked to fill it in. In total, 446 participants from the healthcare organisation (28.3% response) and 561 participants from the financial organisation (47.8% response) completed the questionnaire.

Respondents were asked whether they considered themselves a family caregiver. For this purpose, we added a detailed description in the questionnaire: ‘You are an family caregiver in case you provide long‐term and high‐intensity care for, for example, a chronically ill partner, handicapped child, parent(‐in‐law), friend or neighbour’. In addition, we gave examples of possible caring tasks (for instance help with personal care, domestic help, guidance during hospital visits, help with administration).

In total, 187 employees (18.6% of the total sample) identified themselves as family caregivers, 125 employees in the healthcare organisation and 62 employees in the financial organisation.

### Data collection

The questionnaire consisted of a collection of structured scales to measure indicators of strain, indicators of work and family demands and resources, work–family conflict and work–family enrichment, family–work conflict and family–work enrichment, supplemented with general demographic and employment characteristics. For all scales, mean scores were calculated with higher scores indicating a higher degree or level of the aspect being assessed.

### Demographic and employment characteristics

The demographic and employment characteristics recorded included the following: age (years), gender, contract hours (‘How many hours per week do you work according to your contract?’) and employer (financial or healthcare organisation).

### Caregiver strain

For measuring caregiver strain, we focused on three domain‐specific outcomes: family caregiving‐related ‘subjective’ caregiver burden (39), work‐related emotional exhaustion and a more general rating of mental health. Caregiver burden was measured with the Self‐Rated Burden scale (SRB), consisting of one item ('Can you specify the burden you are experiencing in family care currently?') on a 10‐point scale (0–10) (40). For work‐related emotional exhaustion, that is the core concept of burnout, the five‐item sub‐scale of the Utrecht Burn Out Scale (UBOS) was used (41). The UBOS scale applies a 7‐point frequency scale, ranging from 1 (never) to 7 (always) (e.g. ‘At the end of a working day I feel empty’) (Cronbach’s alpha = 0.87). General mental health was assessed with one item (‘How do you, in general, rate your mental health?’) using a 10‐point scale, ranging from 1 (extremely unhealthy) to 10 (extremely healthy). For purpose of comparability with the interpretation of both other strain variables, the scores on this scale were converted into an ‘ill mental health’ variable.

### Job and family demands

Our independent research variables, that is job and family demands and resources, stem from the JDR model, a model that incorporates the interplay between demands and resources in the job domain (35), and from the work–family interference approach (26) and the model of the work–family interface (28). Workload and work–family conflict were included as job demands and the number of weekly caring hours (as objective measure of care burden (39)), and family–work conflict was designated as family demands.

Workload was measured with five items from the Job Content Questionnaire (JCQ) (42) (e.g. ‘Do you have to work fast?’) (Cronbach’s alpha = 0.54). To identify work–family and family–work conflicts, we used two scales from the SWING (Survey Work‐home Interaction–NijmeGen) (43): (1) work–family conflict, that is negative load reactions developed at work hamper functioning at home, measured with 8 items (e.g. ‘How often does it happen that your work schedule makes it difficult for you to fulfil your domestic obligations?’; Cronbach’s alpha = 0.84); (2) family–work conflict, that is negative load reactions developed at home hamper functioning at work, measured with 4 items (e.g. ‘How often does it happen that it is difficult to concentrate at work, because you are worrying about things in your private situation?’; Cronbach’s alpha = 0.75). Workload, work–family and family–work conflicts were assessed on a Likert scale, ranging from 1 (never) to 4 (always). With the item, ‘On average, how many hours per week do you spend on family care’, the number of hours of family care was assessed.

### Job and family resources

Resources in our model include autonomy and social support from supervisor and colleagues (derived from the JDR model (34)) and work–family and family–work enrichment (based on the work–family perspective (26)). The JCQ (42) was used to assess autonomy with five items on a 4‐point Likert scale (e.g. ‘Can you decide how to do your work?’; Cronbach’s alpha = 0.77). Supervisor support (e.g. ‘My supervisor has an eye for employees’ well‐being’; Cronbach’s alpha = 0.83) and colleague support (e.g. ‘My colleagues give me a hand to get the work done’; Cronbach’s alpha = 0.80) were measured with four items on a 5‐point Likert scale.

To identify work–family and family–work enrichment, we used two scales from the SWING (43): (1) work–family enrichment, that is positive load reactions developed at work facilitate functioning at home, was measured with 5 items (e.g. ‘How often does it happen that you come home cheerfully after a successful day at work, positively affecting the atmosphere at home?’; Cronbach’s alpha = 0.75); (2) family–work enrichment, that is positive load reactions developed at home facilitate functioning at work, was measured with 5 items (e.g. ‘How often does it happen that after a nice weekend with partner/family/friends you enjoy doing your work?’; Cronbach’s alpha = 0.81. For both variables, a 4‐point Likert scale was used ranging from 1 (never) to 4 (always) (43).

### Ethical considerations

Approval was provided by the appropriate review committees from both organisations, and participants were obliged to provide written consent before participation. All information was anonymised and treated as confidential. Participants were permitted to withdraw at any time for personal reasons after starting the study. The necessary information on study content and procedures was given on the organisational intranet and by supervisors.

### Data analysis

SPSS 24.0 (44) was used to conduct the data analysis. There were no missing data as we set a ‘no missing allowed’ constraint on our online survey. First, descriptive analyses were performed. Pearson correlations were calculated to gain an initial insight into the intercorrelations between the research variables and to check for multicollinearity. Secondly, we examined the residuals of the regression analyses for the three outcome variables (i.e. caregiver burden, work‐related emotional exhaustion and ill mental health) to test for the assumptions of linearity, homoscedasticity, independence and normality (45). The results showed that these assumptions for regression were all met. Due to skewness, a log‐transformation had to be performed for all three outcome variables. Finally, a multiple hierarchical regression analysis was conducted in three steps. At step one, demographic and employment characteristics were entered into the regression model, at step two the job and family demands, and finally the job and family resources at step three. In this study, we used a statistical significance level of p ≤ 0.05.

## Results

Of the 187 participants, most were women (81.8%) and worked at the healthcare organisation (67%). Overall, ages ranged between 24 and 64 years (mean = 47.5, SD 8.6), and the number of contract hours varied between 3 and 40 hours per week (mean = 26.7, SD 8.9) (Table 1). The average number of caregiving hours was 9.9 hours per week, with 45.5% providing care ≤5 hours, 35.8% 6–14 hours and 18.7% ≥15 hours. Significant differences between the healthcare and financial organisation were found for sex (in care organisation 90.4% female and in financial organisation 64.5%) (Χ^2^ = 18.7; p = 0.0001), contract hours (in care organisation 23.3 hours/week and in financial organisation 33.4 hours/week) (t = 8.7; p = 0.0001), and family–work enrichment (in care and financial organisation the mean score was, respectively, 2.6 and 2.1) (t = −3.9; p = 0.0001).

**Table 1:** Means, standard deviations and intercorrelations of the research variables (*n* = 187)

	M	SD	1	2	3	4	5	6	7	8	9	10	11	12	13	14
Age	47.48	8.64	1													
Contract hours	26.67	8.85	−0.23**	1												
Hours of informal care	10.20	13.15	−0.04	−0.05	1											
Workload	2.40	0.49	−0.17*	0.21**	0.05	1										
WF conflict	1.69	0.42	−0.16*	0.23**	0.13	0.37**	1									
FW conflict	1.41	0.34	−0.14	0.17*	0.42**	0.12	0.36**	1								
Autonomy	2.78	0.53	0.03	0.19**	0.02	−0.02	−0.03	0.03	1							
WF enrichment	2.13	0.68	0.14	−0.07	0.02	−0.05	0.03	−0.12	0.16*	1						
FW enrichment	2.44	0.80	0.02	−0.22**	0.04	−0.01	−0.04	−0.11	−0.07	0.61**	1					
Supervisor support	3.00	0.55	0.01	0.04	−0.05	−0.08	−0.16*	−0.07	−0.07	0.21**	0.13	1				
Colleague support	3.24	0.52	−0.04	0.02	0.04	0.03	−0.02	0.05	0.05	0.01	0.07	0.13	1			
Emotional exhaustion	2.56	1.09	−0.07	0.07	0.13	0.28**	0.56**	0.35**	−0.10	0.01	−0.07	−0.16*	−0.07	1		
Ill mental health	2.11	1.19	−0.10	0.03	0.20**	0.16*	0.35**	0.36**	0.01	−0.05	−0.12	−0.18*	−0.17*	0.58**	1	
Caregiver burden	4.54	2.29	−0.01	0.07	0.39**	0.12	0.31**	0.41**	0.08	0.01	−0.03	0.01	0.01	0.22**	0.27**	1

* p ≤ 0.05.

** p ≤ 0.01.

There were no significant correlations between the demands and resources variables, except for the association between work–family conflict and supervisor support (Table 1). Work‐related emotional exhaustion showed significant positive correlations with workload, work–family conflict and family–work conflict, as well as a negative correlation with supervisor support. Ill mental health was significantly positively correlated with workload, work–family conflict, family–work conflict and hours of family caregiving, and negatively with supervisor support and colleague support. Caregiver burden was significantly positively related with hours of family caregiving, work–family conflict and family–work conflict. The rather high intercorrelation between work–family and family–work enrichment (=0.6) was not expected to influence our regression results, as both variables were not significantly related to the three outcome variables.

Table 2 presents the results of the three hierarchical linear regression analyses. Family caregiver burden, work‐related emotional exhaustion and general ill mental health have more variance in common with job and family demands (work‐related emotional exhaustion 34%; ill mental health 12%; and caregiver burden 16%, respectively) than with job and family resources (work‐related emotional exhaustion 1.2%; ill mental health 6.2%; caregiver burden 1.4%, respectively).

**Table 2:** Hierarchical linear regression results for three indicators of strain: work‐related emotional exhaustion, general ill mental health and subjective caregiver burden (n = 187)

	Emotional exhaustion		Ill mental health		Caregiver burden	
	B	β	B	β	B	β
Demographic/employment characteristics						
Gender	0.04	0.08	−0.19	−0.06	0.05	0.06
Age	0.00	−0.11	−0.01	−0.05	−0.00	−0.02
Contract hours	0.00	0.07	0.02	0.11	0.00	0.10
Employer	0.04	0.11	0.21	0.08	0.04	0.06
*R* ^2^	0.02		0.01		0.02	
Δ*R* ^2^	0.02		0.01		0.02	
Demands						
Hours of informal care	−0.00	−0.04	0.01	0.10	0.01	0.25**
Workload (1–4)	0.05	0.14**	0.31	0.13	0.02	−0.03
WF conflict (1–4)	0.20	0.48**	0.56	0.19**	0.17	0.24**
FW conflict (1–4)	0.09	0.18**	0.54	0.14*	0.14	0.15*
*R* ^2^	0.36		0.13		0.21	
Δ*R* ^2^	0.34**		0.12**		0.16**	
Resources						
Autonomy (1–4)	−0.01	−0.03	0.15	0.07	0.04	0.07
WF enrichment (1–4)	0.03	0.11	0.09	0.05	0.02	0.01
FW enrichment (1–4)	−0.01	−0.06	−0.06	−0.04	−0.02	−0.04
Supervisor support (1–5)	−0.02	−0.06	−0.40	−0.18**	0.05	0.09
Colleague support (1–5)	−0.02	−0.03	−0.34	−0.14**	−0.02	−0.04
*R* ^2^	0.37		0.19		0.23	
Δ*R* ^2^	0.01		0.06**		0.01	

* p ≤ 0.01.

** p ≤ 0.001.

As can be seen, higher levels of work–family conflict and of family–work conflict were associated with higher levels of all three indicators of strain, so as well as with work‐related emotional exhaustion, poor mental health and with subjective caregiver burden. No significant associations were found for the resources we studied, neither for work–family enrichment nor for family–work enrichment.

Employed caregivers who experienced more support from their supervisor or colleagues scored lower on ill mental health. A higher workload was associated with a higher level of work‐related exhaustion. Caregiver burden was higher for those who provided more hours of family care.

## Discussion

In this study, we focused on exploring three domain‐specific forms of perceived strain in family caregivers with a paid job: caregiver burden, ill mental health and work‐related emotional exhaustion. We surveyed the relationship between caregiver strain and demands and resources, not only in the work domain, but also within the domain of the family, and joined the latest understandings within the work–family interference approach about the coexistence of conflict and enrichment processes (26).

Some of our outcomes were as expected and confirm those reported earlier, albeit from studies within different populations. In our study among employed family caregivers, work‐related emotional exhaustion was associated with higher workload. The strong and consistent relationship between this core concept of burnout and workload was already confirmed, for instance for professional healthcare workers (46, 47).

Family caregivers with more supervisor and colleague support at their work experienced lower levels of ill mental health, as was also reported earlier. In a study among 1236 employees working in home furniture (48), a positive correlation between both supervisor and colleague support with mental health was found. Lack of supervisor support turned out to be a significant risk factor for the two ill mental health measures (i.e. stress feelings and burnout symptoms) used in a large study (*n* = 6091) among insurance, banking, transportation and healthcare personnel (49). In another study, supervisor and colleague support were demonstrated to help family caregivers to maintain their career perspective while caring for care recipients (50).

‘Subjective’ caregiver burden, considered as the caregivers‘ attitude towards or emotional reaction to the caregiving experience, being the product of a specific, interpretative process (39), was observed to be associated with more hours of family care, which was also in line with results from earlier studies (e.g. 51). ‘Objective’ burden relates to the scope of help to care recipients, for example the total amount of caregiving hours (39). In our conceptual model, we took the number of caregiving hours as a demand stemming from the family caregiving situation.

Remarkably, all three strain indicators (caregiver burden, ill mental health and work‐related emotional exhaustion) were associated with higher demands of both work–family conflict and family–work conflict, so work demands spilled over to the family domain and vice versa, lending support to the cross‐domain hypothesis (28), but, at the same time, stayed in the same domain, supporting the matching hypothesis (29). These associations were observed for both domain‐specific outcomes, as well as for the general outcome of ill mental health. It seems that in our population demands from both domains are so high, beyond certain limits, that no clear distinction in the direction of spillover could be observed (33). Noteworthy, in a study among haematologists and oncologists working in private practice (52), both work–family conflict and family–work conflict were positively associated with work‐related exhaustion, showing that also in other populations a clear distinction in the domain‐specific direction of the conflict, from work to family or from family to work, is sometimes not visible.

In contrast, no notable associations were found between caregiver’s strain outcomes and both resources of work–family and family–work enrichment. This corresponds with the results of a recent survey in the Netherlands that suggested that higher work–family and family–work conflicts are associated with higher levels of both fatigue and health complaints, whereas no correlations between these health indicators and both enrichment factors could be demonstrated (53). Moreover, there is some evidence from other studies that informal social support (e.g. from family members and friends) is associated with lower caregiver burden (54, 55). Unfortunately, we did not measure social support in the home situation as a potential resource.

In organisational psychology research, autonomy is generally seen as a job resource with a monotone positive relationship with desired outcomes such as well‐being (56), lower scores on burnout, job satisfaction and ill mental health (57, 58, 59).

Interestingly, for our sample of family caregivers, we were not able to confirm this. It is difficult to find a tight explanation for this unexpected result. However, work design scientists suggest that autonomy increases employees’ responsibility for work (60, 61, 62, 63). It could be possible that employed family caregivers have less need for or even avoid job autonomy when it goes along with higher job responsibilities that may be hard to combine with the caregiving demands at home. In addition, the majority of our family caregivers had a part‐time job (mean of contract hours is 26.7 hours), and part‐time work is known to be related to lower levels of autonomy (64).

All‐in‐all, the evidence of the larger impact of demands compared to resources on our strain outcomes is in line with results from other studies among employed family caregivers (34, 57).

## Limitations

Our results should be viewed in the light of some study limitations. First, our data were collected in 2011. However, we do not expect that since then the challenge to combine work with family care has changed. Although a growing number of organisations implemented some kind of personnel policy targeting the needs of employed family caregivers, there has not yet been an extensive evaluation of the effects of these measures. Furthermore, the design of this study was cross‐sectional, which precludes the possibility of measuring change over time and establishing a causal relationship. Moreover, the relatively low response rate, that was substantially lower in the healthcare organisation (28.3%) compared to the financial organisation (47.8%), may have affected the internal and external validity and restricted the generalisability of the findings. Self‐selection bias could be a problem as employees in both organisations identified themselves as family carer.

In addition, the relatively low scores on the three strain indicators could be an indication of another selection effect, as those perceiving higher strain either may not have had the time/energy to respond to the online survey or had already stopped working. Indications are that it becomes difficult to combine family care with employment when individuals provide 15 or more hours of weekly care (2). Only 18.7% of our family caregivers provided at least 15 hours of care per week, which could mean that some high‐intensity carers had already left the job.

The rather strong correlation between work‐related emotional exhaustion and ill mental health (=0.58) might suggest that both data collection scales might have measured overlapping concepts, although emotional exhaustion is explicitly questioned in the work‐related context while mental health is inquired about in a general sense.

In our sample of employed family caregivers, a higher percentage was working in the healthcare organisation (67%) than in the financial organisation (33%). This reflects the fact that in the Netherlands workers in healthcare settings are more likely to provide family care than workers with no experience in a health profession (3 Klerk de). Unfortunately, due to the limited number of respondents, we were not able to study interaction effects between (job and family) demands and (job and family) resources on the outcome variables. This could have helped to investigate whether resources can buffer the impact of demands on caregiver’s strain.

## Conclusion

Family caregivers continue to play a prominent role as a major safety net to meet the needs of chronically ill or disabled relatives or close friends. It remains important to explore and understand their situation and provide us with information that can be helpful to improve their well‐being. In this study, we explored not only the impact of job demands and resources but also the impact of family demands and resources on indicators of employed family caregiver’s strain, that is work‐related emotional exhaustion, ill mental health and subjective caregiver burden.

The results showed that the job demands (workload, work–family conflict) and family demands (family care hours, family–work conflict) have stronger associations with caregiver strain than job and family resources. Demands from both domains, work as well as family, were associated with higher strain levels in the same domain, but also spilled over to the other domain. Both resources of work–family and family–work enrichment and the potentially stimulating job characteristic ‘autonomy’ did not contribute to a lower level of experienced strain. Only the supervisor and colleague support confirmed the predicted relationship with ill mental health.

Until now, the experiences and support needs and measures of the general family caregiver group, whether or not caring for patients with different kinds of diseases, have been examined extensively. However, special attention should be paid to the caregiver who is struggling with combining responsibilities in different domains, at work and at home. Exploring the potential buffering role of resources might reveal how these can be helpful to support employed family caregivers. Moreover, it might be worthwhile to further explore what causes the absence of the assumed positive role of autonomy for employed family carers. Does family caring go with less autonomy, possibly because of avoiding too much responsibility at one’s work? Alternatively, do family caregivers pre‐eminently lack autonomy due to them mainly being a part‐timer?

In the search of adequate resources, it is best to compose a set of measures that provides support not only at work but also in the home situation. In this, employers of the caregivers and health and community care professionals should join forces to customise support to specific features of a family caregiver’s situation.

## Conflict of interest

No conflict of interest has been declared by the authors.

## Author contribution

All authors of this manuscript have agreed on the final version and meet at least one of the following authorship criteria according to the latest guidelines of the International Committee of Medical Journal Editors (ICMJE): (1) substantial contributions to conception and design, acquisition of data, or analysis and interpretation of data; and (2) drafting the article or revising it critically for important intellectual content.

## Ethical approval

Approval was provided by the appropriate committees from both companies, and participants were obliged to provide written consent before after thorough explanation of the procedures.

## Funding

This research received no specific grant from any funding agency in the public, commercial or not‐for‐profit sectors.
